# Incidence trends and risk prediction nomogram of metachronous second primary lung cancer in lung cancer survivors

**DOI:** 10.1371/journal.pone.0209002

**Published:** 2018-12-17

**Authors:** Zhi Gang Hu, Wen Xin Li, Yu Shu Ruan, Fan Jun Zeng

**Affiliations:** 1 Respiratory Disease Research Institute of China, The First College of Clinical Medical Science, Three Gorges University, Yichang, China; 2 Department of Respiratory Medicine, Yichang Central People's Hospital, Yichang, China; University of Zurich, SWITZERLAND

## Abstract

**Background:**

This study was designed to estimate the trends in 5-year incidence of metachronous second primary lung cancer(SPLC) and to establish a risk prediction model to identify candidates who were at high risk of developing metachronous SPLC.

**Methods:**

Incidence data between 2004 and 2007 were obtained from SEER database, including 42453 participants who survived ≥ 2 years after the initial diagnosis of lung cancer. Joinpoint regression analysis was used to calculate the 5-year incidence rates of metachronous SPLC per 100 000 population. Related risk factors of the survivors who developed MSPLC during five years were identified through logistic regression analysis, followed by establishment of risk prediction nomogram. Discrimination (C-index), calibration and decision analysis were further performed to assess the validation and clinical net benefit of risk prediction nomogram.

**Results:**

A total of 1412 survivors with lung cancer developed MSPLC during five years, with 3546 per 100 000 population of age-adjusted 5-year incidence. Age, histology, tumor stage, and radiation were recognized as risk factors of metachronous SPLC, as indicated by logistic regression analysis. The risk prediction nomogram of metachronous SPLC harbored moderate discrimination(C-index = 0.67) and good calibration, with the risk of 0.01 to 0.11.The decision curve analysis showed that clinical net benefit of this risk prediction nomogram in a range of risk thresholds (0.01 to 0.06) was higher compared to all-screening or no-screening strategies.

**Conclusions:**

Collectively, the cumulative risk of metachronous SPLC of the survivors increased over time. The risk prediction nomogram was available to select high-risk survivors who should regularly undergo computed tomography screening.

## Introduction

Based on recent epidemiological surveys, lung cancer has become one of the most common carcinomas with high mortality[1,2]. With the promotions of screening programs and the development of therapeutic approaches for lung cancer, the number of long-time survivors, especially those with early stage lung cancer, is gradually increasing. In spite of the improvement of survival time and the reduction of overall mortality, the increased risk of second primary lung cancer (SPLC) among lung cancer survivors can not be neglected. Compared with the population without lung cancer history, the risk of developing SPLC among survivors with initial primary lung cancer (IPLC) increases by four- to sixfold[3].The prognosis of SPLC will become increasingly worse in the case of more advanced stage at diagnosis, similar to IPLC. However, excessive radiation exposure for lung cancer screening may increase the risk of lung cancer and other major cancers, especially in women aged 50–54 years old[4].This finding indicated that low-dose computed tomography surveillance for SPLC may be associated with increased risk of second primary cancer. Risk prediction models based on risk factors of SPLC can not only effectively select high-risk survivors who should receive screening, but also decrease radiation exposure of low- risk lung cancer survivors.

Various risk prediction models of IPLC have been developed, including two risk models with good discrimination(C-index>0.8)[5–8]. In these two models, both personal history of cancer and family history of lung cancer are regarded as important risk factors of IPLC[5,7].These results suggest that some cancer-related genetic driver mutations seemingly play an important role in the tumorigenesis and progression of lung cancer. Therefore, the survivors of IPLC are at a higher risk of developing SPLC. Han and his colleagues[9] found that the 6-year risk of developing lung cancer among the IPLC patients who survived ≥ 5 years is fourfold higher than those who had no history of lung cancer. However, only few studies paid attention to the identification of the risk factors of SPLC and subsequent establishment of a risk prediction model for SPLC[10–12]. Smoking is an indisputable risk factor of IPLC, however, whether smoking increases the risk of SPLC still remains controversial[10,13–15]. The first risk prediction model of SPLC is focused on IPLC patients who survived ≥ 5 years, which includes three risk factors, namely age, histology and the extent of disease[9]. Based on these three risk factors, the median 10-year risk of SPLC is calculated as 8.35% (range, 0.59% to 14.3%) using the risk model.

The favorite diagnostic criterion of metachronous SPLC was originated from the proposal of Martini and Melamed[16]. The patients who are diagnosed with metachronous SPLC must fulfill any one of the following two criteria: 1)There are different histological types between IPLC and SPLC; 2) When IPLC and SPLC harbor the same histology, they must have disease-free interval of at least 2 years, or origin from carcinoma in situ, or occur in different lobes with no metastatic carcinoma of common lymph nodes and no extra-pulmonary metastasis at the time of diagnosis. In the present study, we adopted this diagnostic criterion and estimated the 5-year incidence trends of developing metachronous SPLC in IPLC patients who survived ≥ 2 years through Joinpoint regression analysis. Logistic regression analysis was used to identify the risk factors of MSPLC. Moreover, we established a risk prediction nomogram of metachronous SPLC based on these risk factors and evaluated its validity through discrimination and calibration. Finally, a decision curve analysis was performed to identify the potential clinical benefit of our prediction nomogram.

## Methods

### Study data

The incidence data of LC were obtained using the population-based SEER-18 database between 2004 and 2007. The study participants were limited to IPLC patient who survived ≥ 2 years. The following information must be included in all participants: accurate time of diagnosis(year and month), survival time, and positive diagnostic information. Histological classification was in accordance with the 3rd edition of the International Classification of Diseases of Oncology. The participants whose histology were malignant neoplasm(8000/3), or malignant tumor cell (8001/3),carcinoma (8010/3), or non small cell lung cancer (8046/3) were not incorporated into this study. Lung cancer was roughly divided into five categories, including adenocarcinoma, squamous cell carcinoma, large cell carcinoma, small cell carcinoma, and other. The IPLC stage of patients was determined based on the 6th edition of the AJCC’s Cancer Staging Manual. All eligible participants were followed-up for 5 years, until the diagnosis of a new primary cancer, until death, or the end of follow-up, whichever occurred first. We collected the following demographic variables: Age(< 50 years, 50 to 59 years, 60 to 69 years, 70 to 79 years, ≥80 years), Sex(male, female), Race(white, black, Asian or Pacific Islander, American Indian/Alaska Native, unknown), Rural-Urban Continuum Code 2013(counties, comp rural, urban, unknown),Marital status at diagnosis(married, unmarried, unknown), Histology (adenocarcinoma, squamous cell carcinoma, large cell carcinoma, small cell carcinoma, other), Grade(well, moderately, poorly, undifferentiated, unknown), Tumor size(<3.5cm, 3.5cm to 6.9cm, ≥7.0cm, unknown), Regional nodes positive(0, 1, 2, ≥ 3, unknown), Condensed stage(CS) extension(T0, T1, T2, T3, T4, unknown), CS lymph nodes(N0, N1, N2, N3, unknown), CS metastasis(M0, M1, unknown), Extent of IPLC(Derived summary stage 2000:localized, regional, distant, and unknown), Tumor stage(I, II, III, IV, unknown),Radiation(yes, no, unknown), Surgery to Primary Site(no surgery, pneumonectomy, lobectomy/bilobectomy, other surgery, unknown), Chemotherapy(yes, no/unknown), diagnostic year and month, survival time, and diagnostic information.

### Statistical analysis

#### Incidence trends of metachronous SPLC

The age-standardized cancer incidence per 100,000 based on the same standard population can effectively reduce the potential confounding effect of age[17]. In our study, age was subdivided into 5 groups: < 50 years, 50 to 59 years, 60 to 69 years, 70 to 79 years, and ≥80 years. Standard population is defined as the patients who were diagnosed between 2004 and 2007 and had positive histology and accurate time of diagnosis(year and month). We determined the incidence of metachronous SPLC of all study participants after a 5-year follow-up. In addition, metachronous SPLC was divided into two categories: MSPLC with different histology and metachronous SPLC with same histology. Cumulative incidence was calculated by dividing the observed number of MSPLC cases until 1, 2, 3, 4, and 5 years of follow-up by the total number of survivors in the beginning of the study. In addition, partial participants possibly died of other causes (such as cardiovascular and cerebrovascular diseases) before developing SPLC. In consideration of the competing risk of all-cause death during the 5-year follow up, we simultaneously obtained unbiased estimates of the risk of SPLC by removing dead participants at the end of 1, 2, 3, 4, and 5 years of follow-up.

The Surveillance Research Program of the US National Cancer Institute suggests using the Joinpoint software to calculate age-adjusted cancer incidence and analyze cancer incidence trends[17].The value of annual percentage change(APC) was used to measure the magnitude of cancer incidence trends for each segment or time period[18, 19]. Bayesian approach was proposed to estimate APCs from age-adjusted cancer incidence data[19]. *P* value of APC was calculated according to a t distribution. A *P* value less than 0.05 indicated a two-sided statistical significance.

#### The development and validation of risk prediction nomogram

Related risk factors of metachronous SPLC were identified through multivariate logistic regression analysis with backward model-selection procedure. The odds ratio (OR) was used to describe the clinically significant effects of metachronous SPLC risk factors The following variables were incorporated into this logistic regression analysis: Age, Sex, Race, Rural-Urban Continuum Code 2013, Marital status at diagnosis, Histology, Grade, Tumor size, Regional nodes positive, CS extension, CS lymph nodes, CS metastasis, Extent of IPLC, Tumor stage, Radiation, Surgery to Primary Site, and Chemotherapy. The problem of collinearity of all variables was assessed through tolerance and variance inflation factor. If tolerance of variable was less than 0.1 and variance inflation factor was greater than 5, this variable may be considered to be removed from this study. When the *P* value of the variable was less than 0.05, this variable was considered as a risk factor of metachronous SPLC. Additionally, interaction terms of risk factors of developing SPLC were also evaluated. All related risk factors of metachronous SPLC were used to develop its corresponding risk prediction nomogram based on the multivariate logistic model. Each risk factor had a score on the points scale, which can be used to estimate the impact on metachronous SPLC. By determining the score of each risk factor and calculating the total score, clinicians can estimate the risk of participants developing metachronous SPLC. The performance of the risk prediction nomogram was measured through discrimination (C-index) and calibration(calibration curve)[20]. Subsequently, internal validation was performed to further test the nomogram’s performance. The value of the C-index statistic ranged from 0.5 (no discrimination) to 1 (perfect discrimination), and higher C-index values indicated a better prediction model[20].Bootstraps with 1000 resamples were used to decrease overfit bias.

#### Clinical usefulness of risk prediction nomogram

Clinical usefulness was the last component in the evaluation of nomogram performance, which was supposed to determine whether nomogram-assisted decisions effectively improved the outcome of patients[20]. Decision analysis curve and clinical impact curves were proposed to assess the clinical usefulness of the risk prediction nomogram. The decision analysis illustrated the net benefit against the threshold probability with graphical curve, which can facilitate clinicians in implementing a medical intervention. The clinical impact curve provided a visual representation of the estimated number who would be deemed to be at high risk with different threshold probability and the number of true positives among 1,000 participants[21].

Joinpoint software, SPSS software, and R software were used to complete the above-mentioned analyses. R package ‘rms’ was adopted to complete the development and validation of risk prediction nomogram. Decision analysis of risk prediction nomogram was performed through R package ‘rmda’. This study was deemed exempt for review by the Institutional Review Board at China, Three Gorges University.

## Results

### Clinical characteristics

SEER data between 2004 to 2007 included 134915 IPLC patients with positive histology and accurate time of diagnosis(year and month), who were considered as standard population of this study. We identified 42453 participants who survived ≥ 2 years after the diagnosis of IPLC. During the 5-year follow-up, there were 22818 participants who died and 1412 participants who developed metachronous SPLC. The survivors with metachronous SPLC included 1071 metachronous SPLC with different histology and 341 metachronous SPLC with the same histology. Adenocarcinoma (54.3%) and squamous cell carcinoma(28.2%) were the main histological classification of metachronous SPLC, similar to IPLC. In addition, 96.2% of metachronous SPLC with same histology comprised adenocarcinoma(61.0%) and squamous cell carcinoma(35.2%). In total, 87 IPLC patients with small cell carcinoma and 53 IPLC patients with large cell carcinoma developed metachronous SPLC. However, among these patients, only one patient with large cell carcinoma developed metachronous SPLC with same histology among these patients. The patients aged 60–79 years(64.5%) accounted for the main population of IPLC, who seemingly harbored higher risk of metachronous SPLC(73.2%). The proportion of stage I IPLC was 46.2% in study participants, while its proportion was 69.8% in metachronous SPLC, including 684 patients who had different histology(63.9%) and 302 patients who had same histology (88.8%).The patients undergoing radiation treatment were associated with lower risk of metachronous SPLC compared with those who did not receive radiation treatment(2.3% vs 4.4%, p< 0.001). In the IPLC patients with radiation treatment, there were 238 patients of developing metachronous SPLC with different histology (22.2%, 238/1071) and 26 patients of developing metachronous SPLC with same histology(7.6%, 26/341), respectively. All detailed characteristics were listed in Table 1.

**Table 1:** Patient characteristics of the study population in SEER.

	Total	MSPLC	MSPLC with different histology	MSPLC with same histology
Age group				
< 50 years	2905 (6.8%)	54 (3.8%)	41 (3.8%)	13 (3.8%)
50 to 59 years	7574 (17.8%)	217 (15.4%)	162 (15.1%)	55 (16.1%)
60 to 69 years	13871 (32.7%)	550 (39.0%)	429 (40.1%)	121 (35.5%)
70 to 79 years	13481 (31.8%)	483 (34.2%)	364 (34.0%)	119 (34.9%)
≥ 80 years	4622 (10.9%)	108 (7.6%)	75 (7.0%)	33 (9.7%)
SEX				
Female	22937 (54.0%)	744 (52.7%)	559 (52.2%)	185 (54.3%)
Male	19516 (46.0%)	668 (47.3%)	512 (47.8%)	156 (45.7%)
Marital status				
Married	24653 (58.1%)	900 (63.7%)	688 (64.2%)	212 (62.2%)
Unmarried	16577 (39.0%)	477 (33.8%)	356 (33.2%)	121 (35.5%)
Unknown	1223 (2.9%)	35 (2.5%)	27 (2.5%)	8 (2.3%)
Race				
Asian or Pacific Islander	2568 (6.0%)	51 (3.6%)	41 (3.8%)	10 (2.9%)
American Indian /Alaska Native	156 (0.4%)	6 (0.4%)	5 (0.5%)	1 (0.3%)
Black	3799 (8.9%)	108 (7.6%)	83 (7.7%)	25 (7.3%)
White	35859 (84.5%)	1247 (88.3%)	942 (88.0%)	305 (89.4%)
Unknown	71 (0.2%)	0 (0.0%)	0 (0.0%)	0 (0.0%)
Regional distribution				
Counties	36890 (86.9%)	1220 (86.4%)	921 (86.0%)	299 (87.7%)
Urban	4830 (11.4%)	167 (11.8%)	132 (12.3%)	35 (10.3%)
Comp rural	694 (1.6%)	22 (1.6%)	15 (1.4%)	7 (2.1%)
Unknown	39 (0.1%)	3 (0.2%)	3 (0.3%)	0 (0.0%)
Tumor size				
< 3.5cm	23819 (56.1%)	913 (64.7%)	671 (62.7%)	242 (71.0%)
3.5 to 6.9 cm	11091 (26.1%)	352 (24.9%)	275 (25.7%)	77 (22.6%)
≥ 7.0 cm	2353 (5.5%)	58 (4.1%)	48 (4.5%)	10 (2.9%)
Unknown	5190 (12.2%)	89 (6.3%)	77 (7.2%)	12 (3.5%)
T				
T0	130 (0.3%)	2 (0.1%)	2 (0.2%)	0 (0.0%)
T1	14997 (35.3%)	626 (44.3%)	456 (42.6%)	170 (49.9%)
T2	14074 (33.2%)	527 (37.3%)	395 (36.9%)	132 (38.7%)
T3	1681 (4.0%)	42 (3.0%)	35 (3.3%)	7 (2.1%)
T4	6750 (15.9%)	143 (10.1%)	121 (11.3%)	22 (6.5%)
Unknown	4821 (11.4%)	72 (5.1%)	62 (5.8%)	10 (2.9%)
Positive nodes of IPLC				
0	19286 (45.4%)	960 (68.0%)	670 (62.6%)	290 (85.0%)
1	2817 (6.6%)	57 (4.0%)	57 (5.3%)	0 (0.0%)
2	1262 (3.0%)	18 (1.3%)	18 (1.7%)	0 (0.0%)
≥3	1645 (3.9%)	31 (2.2%)	31 (2.9%)	0 (0.0%)
Unknown	17443 (41.1%)	346 (24.5%)	295 (27.5%)	51 (15.0%)
N				
N0	26592 (62.6%)	1157 (81.9%)	816 (76.2%)	341 (100.0%)
N1	4208 (9.9%)	69 (4.9%)	69 (6.4%)	0 (0.0%)
N2	8087 (19.0%)	144 (10.2%)	144 (13.4%)	0 (0.0%)
N3	1664 (3.9%)	22 (1.6%)	22 (2.1%)	0 (0.0%)
Unknown	1902 (4.5%)	20 (1.4%)	20 (1.9%)	0 (0.0%)
M				
No	35165 (82.8%)	1337 (94.7%)	996 (93.0%)	341 (100.0%)
Yes	5839 (13.8%)	54 (3.8%)	54 (5.0%)	0 (0.0%)
Unknown	1449 (3.4%)	21 (1.5%)	21 (2.0%)	0 (0.0%)
Histology				
Adenocarcinoma	22807 (53.7%)	767 (54.3%)	559 (52.2%)	208 (61.0%)
Squamous carcinoma	10722 (25.3%)	398 (28.2%)	278 (26.0%)	120 (35.2%)
Large cell carcinoma	1478 (3.5%)	53 (3.8%)	52 (4.9%)	1 (0.3%)
Small cell carcinoma	3285 (7.7%)	87 (6.2%)	87 (8.1%)	0 (0.0%)
Other	4161 (9.8%)	107 (7.6%)	95 (8.9%)	12 (3.5%)
Grade				
Well	4766 (11.2%)	212 (15.0%)	157 (14.7%)	55 (16.1%)
Moderate	12607 (29.7%)	513 (36.3%)	360 (33.6%)	153 (44.9%)
Poor	11324 (26.7%)	388 (27.5%)	290 (27.1%)	98 (28.7%)
Undifferentiate	1755 (4.1%)	56 (4.0%)	53 (4.9%)	3 (0.9%)
Unknown	12001 (28.3%)	243 (17.2%)	211 (19.7%)	32 (9.4%)
Extent of IPLC				
Localized	18104 (42.6%)	835 (59.1%)	587 (54.8%)	248 (72.7%)
Regional	14958 (35.2%)	462 (32.7%)	375 (35.0%)	87 (25.5%)
Distant	8497 (20.0%)	101 (7.2%)	96 (9.0%)	5 (1.5%)
Unknown	894 (2.1%)	14 (1.0%)	13 (1.2%)	1 (0.3%)
Tumor stage				
I	19632 (46.2%)	986 (69.8%)	684 (63.9%)	302 (88.6%)
II	3583 (8.4%)	75 (5.3%)	68 (6.3%)	7 (2.1%)
III	9313 (21.9%)	233 (16.5%)	211 (19.7%)	22 (6.5%)
IV	5700 (13.4%)	54 (3.8%)	54 (5.0%)	0 (0.0%)
Unknown	4225 (10.0%)	64 (4.5%)	54 (5.0%)	10 (2.9%)
Surgery				
No surgery	15014 (35.4%)	232 (16.4%)	216 (20.2%)	16 (4.7%)
Pneumonectomy	1219 (2.9%)	19 (1.3%)	12 (1.1%)	7 (2.1%)
Lobectomy/Bilobectomy	20749 (48.9%)	947 (67.1%)	690 (64.4%)	257 (75.4%)
Other	5293 (12.5%)	211 (14.9%)	150 (14.0%)	61 (17.9%)
Chemotherapy				
No/unknown	25351 (59.7%)	999 (70.8%)	719 (67.1%)	280 (82.1%)
Yes	17102 (40.3%)	413 (29.2%)	352 (32.9%)	61 (17.9%)
Radiation treatment				
No	24499 (57.7%)	1090 (77.2%)	781 (72.9%)	309 (90.6%)
Yes	11657 (27.5%)	264 (18.7%)	238 (22.2%)	26 (7.6%)
Unknown	6297 (14.8%)	58 (4.1%)	52 (4.9%)	6 (1.8%)

MSPLC, Metachronous second primary lung cancer

### Incidence trends of metachronous SPLC

**Fig 1:**
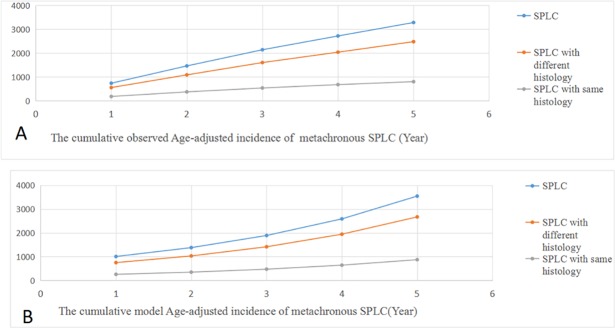
Incidence trends of metachronous second primary lung cancer(SPLC) (A).The cumulative observed age-adjusted incidence of metachronous second primary lung cancer (Year) (B).The cumulative model age-adjusted incidence of metachronous second primary lung cancer with competing risk (Year).

The age-standardized incidence trends of metachronous SPLC are presented in Fig 1. During the 5-year follow-up, the cumulative risk of metachronous SPLC of the survivors gradually increased over time(Table 2). To be specific, the age-standardized 1-year incidence of metachronous SPLC was 737 per 100,000 population, while 5-year cumulative incidence was 3282 per 100,000 population. For metachronous SPLC with different histology, there were 555 per 100,000 population of 1-year incidence and 2479 per 100,000 population of 5-year cumulative incidence. For metachronous SPLC with the same histology, 182 per 100,000 population of 1-year incidence and 802 per 100,000 population of 5-year cumulative incidence were found. Considering the competing risk of all-cause death of IPLC during the 5-year follow up, the age-standardized 1-year incidence and 5-year cumulative incidence of metachronous SPLC were 948 per 100,000 population and 8099 per 100,000 population, respectively. After the 5-year follow up, 6075 per 100,000 population of the survivors might develop metachronous SPLC with different histology, whereas 2024 per 100,000 population potentially developed metachronous SPLC with same histology. The 1-year risk of metachronous SPLC with different and same histology was 713 per 100,000 population and 235 per 100,000 population, respectively.

**Table 2:** Incidence trends of metachronous second primary lung cancer.

The cumulative risk of MSPLC (year)	Observed Age-Adjusted Incidence	Standard Error	Modeled Age-Adjusted Incidence	APC	AAPC
SPLC				36.9^(15.8–61.9)	36.9^(15.8–61.9)
1	737.59	42.17	1007.91		
2	1462.34	59.46	1380.19		
3	2140.58	71.73	1889.97		
4	2717.78	80.79	2588.04		
5	3282.19	88.43	3543.95		
SPLC with different histology				37.3^(16.7–61.6)	37.3^(16.7–61.6)
1	555.34	36.49	750.55		
2	1087.69	51.16	1030.85		
3	1603.03	62.01	1415.82		
4	2038.62	69.77	1944.57		
5	2479.4	76.65	2670.79		
SPLC with same histology				35.7^(13.2–62.7)	35.7^(13.2–62.7)
1	182.25	21.14	257.51		
2	374.65	30.3	349.44		
3	537.55	36.05	474.19		
4	679.16	40.72	643.47		
5	802.79	44.09	873.19		

MSPLC, Metachronous second primary lung cancer

### The development and validation of risk prediction nomogram

**Fig 2:**
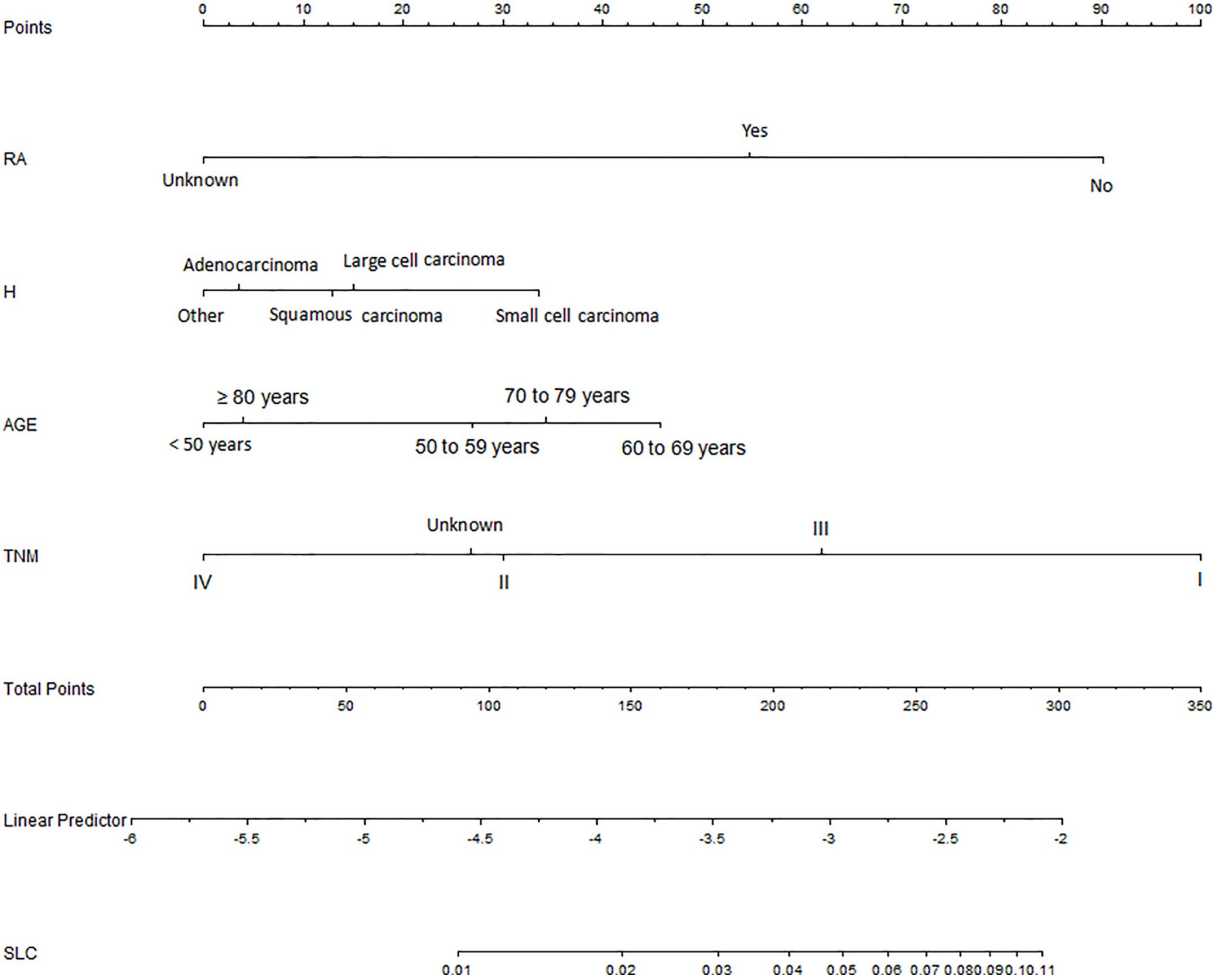
Risk prediction nomogram of metachronous second primary lung cancer.

**Fig 3:**
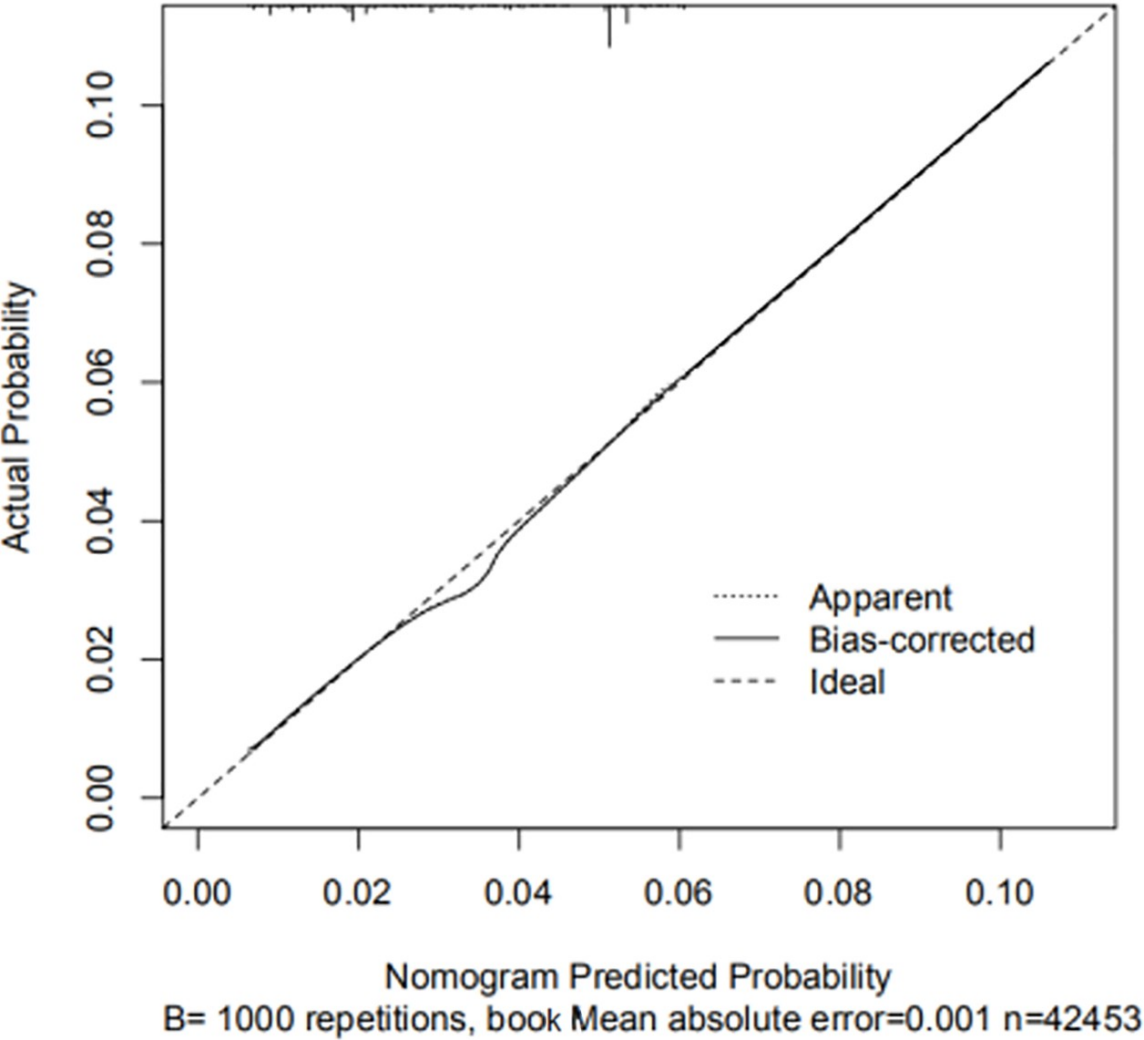
Calibration curve of risk prediction nomogram of metachronous second primary lung cancer.

In univariate analyses, almost all variables were regarded as the related risk factors of developing metachronous SPLC except sex and Rural-Urban Continuum Code 2013. In multivariate logistic regression analyses, histology, age, tumor stage, and radiation were identified as the related risk factors of the survivors who developed metachronous SPLC during the 5-year follow-up(Table 3). No problem of multicollinearity between different variables was found in our study. In addition, a large sample size potentially reduced the risk of multicollinearity. Therefore, we deemed that four risk factors of SPLC harbored stable and meaningful estimation of beta coefficients in our study. There were no interaction terms among age, histology and tumor stage. Compared with IPLC of other histology, the survivors of small cell carcinoma were associated with higher risk of metachronous SPLC (OR = 1.68, 95%CI:1.19–2.37). The risk of metachronous SPLC patients aged 60–69 years was significantly higher than those aged ≥80 years old(OR = 1.60, 95%CI:1.30–1.99). All four risk factors were used to develop the risk prediction nomogram of metachronous SPLC, with the risk of 0.01 to 0.11(Fig 2). Our risk nomogram showed moderate discrimination (C-index = 0.67). In the internal validation, C-index of risk model was still 0.67. Calibration curve indicated good calibration between actual probability and was able to predict the probability of our risk models for predicting metachronous SPLC(Fig 3). In terms of age variable, the patients aged less than 50 or more than 80 years exhibited lower risk of SPLC than those aged 60 to 79 years. In terms of histological variable, the patients with small cell carcinoma had the highest risk of developing metachronous SPLC, followed by large cell carcinoma, squamous cell carcinoma, adenocarcinoma and other histology. Considering the tumor stage, the survivors of IPLC with stage I were associated with prolonged survival time and higher risk of developing metachronous SPLC than other survivors. For survivors with radiation treatment, the risk of developing metachronous SPLC was lower than those without radiation treatment.

**Table 3:** Risk factors of metachronous second primary lung cancer in the final risk prediction nomogram.

	OR	Low	Upper	P
Radiation treatment				
Unknown	reference			
No	1.844	1.255	2.709	0.002
Yes	1.706	1.244	2.338	0.001
Histology				
Other	reference			
Adenocarcinoma	1.011	0.8	1.278	0.927
Squamous carcinoma	1.186	0.925	1.521	0.178
Large cell carcinoma	1.243	0.859	1.799	0.248
Small cell carcinoma	1.679	1.189	2.37	0.003
Tumor stage				
Unknown	reference			
I	2.493	1.421	4.375	0.001
II	1.939	0.992	3.79	0.053
III	2.841	1.541	5.237	0.001
IV	2.651	0.543	12.931	0.228
Age group				
≥ 80 years	reference			
< 50 years	0.943	0.672	1.324	0.735
50 to 59 years	1.295	1.017	1.649	0.036
60 to 69 years	1.605	1.295	1.989	<0.001
70 to 79 years	1.396	1.127	1.73	0.002

MSPLC, Metachronous second primary lung cancer

### Clinical usefulness of risk prediction nomogram

**Fig 4:**
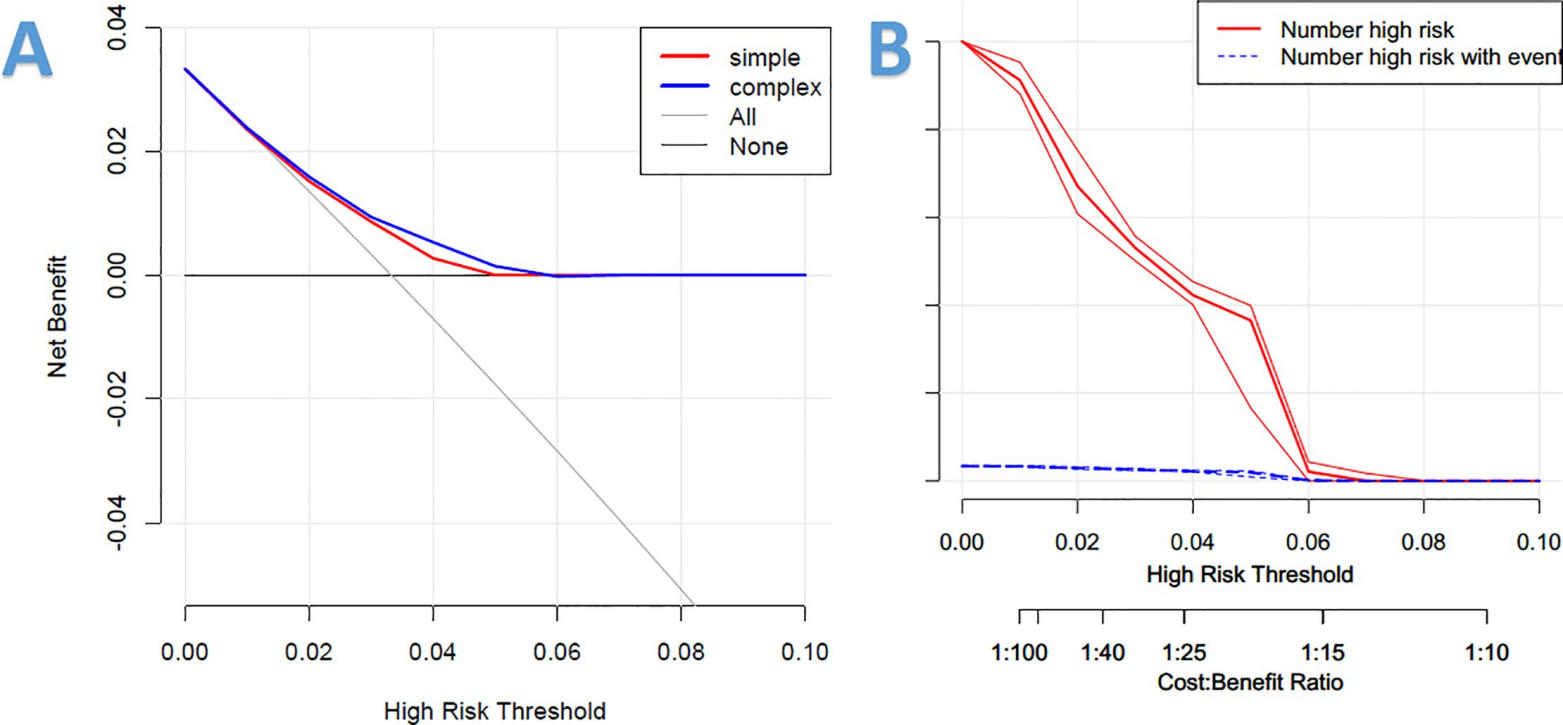
Decision analysis curve and clinical impact curve of risk prediction model. (A)Decision analysis curve(Simple = Radiation treatment; Complex = Risk prediction model); (B)Clinical impact curve.

To evaluate clinical usefulness of our risk prediction nomogram, a decision analysis was performed along with graphical decision curve and clinical impact curve. In comparison with all-screening or no-screening strategies, the risk prediction nomogram with risk threshold from the given interval of 0.01 to 0.06 may obtain more clinical net benefit in the decision curve(Fig 4). In addition, decision analysis curve also demonstrated that radiation treatment with risk threshold of 0.01 to 0.05 potentially yielded clinical net benefit. Clinical impact curve visually showed the estimated numbers who were deemed high risk and true positives in the range of 0.01 to 0.06 by using our risk model. For example, if 1,000 participants were screened with the use of 0.04 risk threshold, 420 participants would be declared with high risk of metachronous SPLC, whereas 20 participants were truly positive. Given the rate of metachronous SPLC is approximately 0.04(20/420), the other 580 participants might avoid unnecessary radiation exposure for surveillance of metachronous SPLC through the clinical utility of our risk nomogram.

## Discussion

In our study, approximately 1.3% and 8.3% of 1-year incidence and 5-year cumulative incidence of SPLC were observed, respectively. During the 5-year follow-up, 2.7% of study participants developed metachronous SPLC with different histology, with 0.7% of 1-year incidence. In consideration of the competing risk of all cause death of IPLC, there were 1.3% of 1-year incidence and 8.3% of 5-year cumulative incidence of metachronous SPLC, respectively. Previous studies demonstrated that the risk of SPLC was 1% to 2% per patient per year and gradually increased over time without plateau, which was consistent with our study[12,22–24]. Han and his colleagues[9] argued that the median 5-year cumulative incidence of developing metachronous SPLC was 5.2% in the presence of competing risks, which was lower than that of our study.

Through multivariate logistic regression analysis, we identified four related risk factors of survivors who developed metachronous SPLC during the 5-year follow-up, including histology, age, tumor stage, and radiation treatment. Our risk prediction nomogram exhibited that small cell lung carcinoma patients had the highest risk of developing metachronous SPLC in all types of lung cancer, which was similar to previous studies. Small-cell lung cancer patients who survived more than 2 years were found to possess a seven- to sixteen-fold higher risk of developing SPLC than a similar population in Canada and the USA[24–27]. However, the risk of IPLC patients who developed SPLC was four- to sixfold higher than the risk of the individuals who developed IPLC[3, 9]. The risk of developing metachronous SPLC of lung adenocarcinoma seemed to be lower than that of lung squamous cell carcinoma[9]. Continuous smoking and radiation treatment were regarded as the feasible interpretations. However, as previously mentioned, the impact of continuous smoking on metachronous SPLC is controversial. Most of studies suggested that continuous smoking after the diagnosis of IPLC is associated with the increased risk of metachronous SPLC [10,13,15]. A recent study by Ripley and his colleagues[14] found no correlation between continued smoking and SPLC. Unfortunately, the SEER database fails to provide a detailed information associating the risk of SPLC and smoking. Hence, we were unable to evaluate the effect of continuous smoking on metachronous SPLC. Whether radiation treatment increased the risk of developing metachronous SPLC was also disputable[11,25,26]. Earlier studies demonstrated that patients with small cell carcinoma undergoing radiation treatment had approximately twofold higher risk of developing SPLC than those without radiation treatment[25,26]. Khanal and his colleagues[11] reported that radiation treatment of IPLC is an independent risk of second primary malignancy and is also associated with decreased risk of second primary malignancy in adenocarcinoma and squamous cell carcinoma. In our study, radiation treatment of IPLC was found to potentially decrease the risk of developing metachronous SPLC through multivariate logistic regression analysis and decision analysis. In addition, radiation treatment with risk threshold from the given interval of 0.01 to 0.05 may obtain more clinical net benefit in the decision curve (Fig 3). However, we also found that small cell carcinoma patients with radiation treatment easily developed MPSLC with different histology, who were likely to be accompanied by higher risk of metachronous SPLC compared with those without radiation treatment(3.1% vs 2.8%). We supposed that radiation treatment potentially increased the risk of metachronous SPLC with the histology of squamous cell carcinoma and adenocarcinoma, while radiation treatment also significantly decreased the risk of developing metachronous SPLC. The clinical benefit of radiation treatment was obviously greater than the harm. In addition, our study population had shorter follow-up time than that of Han and his colleagues. Radiation treatment may decrease the short-time risk of metachronous SPLC, but had no significant impact on the long-time risk of metachronous SPLC. A perplexing phenomenon in our study was that neuroendocrine carcinoma of IPLC, mainly including small cell carcinoma and large cell carcinoma, seemed to easily develop metachronous SPLC with different histology. Whether neuroendocrine-related driver mutations regulate the development of metachronous SPLC with the histology of squamous cell carcinoma and adenocarcinoma deserves further investigation.

Another noteworthy result was that patients aged < 50 years had lower risk of developing SPLC and longer survival time than other patients, especially those aged 60 to 69 years. Han and his colleagues[9] also reported that IPLC patients aged < 50 years had the lowest risk of developing SPLC, while IPLC patients aged 60 to 69 years had the highest risk of developing SPLC. Another study found that IPLC patients aged > 60 years had significantly higher risk of developing SPLC than those aged < 50 years (HR = 4.03, 95%CI:1.26–12.93)[10]. These findings were rather counterintuitive as young patients exhibited longer survival time and were more likely to develop SPLC[9].A potential explanation is that the survivors in different age groups have different histological distributions, which potentially influence the incidence of metachronous SPLC. Patients aged < 50 years had lower risk of developing lung squamous cell carcinoma(12.4% vs 26.3%) and higher risk of developing IPLC of other histology(24.1% vs 8.7%) than those who were diagnosed at 60 to 69 years of age, with similar proportions in the remanent histology. Our risk prediction nomogram displayed the following risk priority: small cell carcinoma, large cell carcinoma, squamous cell carcinoma, adenocarcinoma and other histology. Therefore, the overall incidence of metachronous SPLC among the survivors aged < 50 years may be lower than those with age of 60 to 69 years. In addition, there were more lung adenocarcinoma(57.7% vs 52.8%) and less small cell carcinoma(3.9% vs 8.7%) in IPLC patients with aged ≥ 80 years than those with aged 60 to 69 years, while the proportions of the remanent histologies were similar. Theoretically, the incidence of metachronous SPLC in the survivors with aged ≥ 80 years should be lower compared with those with aged 60 to 69 years, which was confirmed by both our risk prediction nomogram and the results by Han et al [9]. Why different histological distributions of IPLC appear in different age groups and whether potentially different driver mutations influence the development of SPLC remain to be further studied and verified.

Our study had the following strengths. First, the participants of our study came from one of the largest population-based cancer registries in the world. The SEER-18 database enrolls cancer cases from 18 regions of the USA and covers approximately 28% of the U.S. population with high-quality data collection and maintenance, which effectively avoids the selection bias of single-center study and small-sample studies[28]. Second, we not only estimated the age-standardized incidence trends of metachronous SPLC in the IPLC patients who survived ≥ 2 years through Joinpoint regression analysis but also estimated the trends of metachronous SPLC with different histology and with same histology. Third, we established one risk prediction nomogram of metachronous SPLC for the IPLC survivors and further tested its validity with moderate discrimination and good calibration. The decision analysis and clinical impact curves visually showed the clinical net benefit of our risk model with the risk threshold of 0.01 to 0.06. These results indicated that our risk nomogram can assist clinicians in effectively evaluating the risk of IPLC patients in developing SPLC. In addition, we detected the impact of radiation treatment on metachronous SPLC through the multivariate logistic regression analysis and decision analyses, which make our results more convincing and accurate.

However, our study has some limitations. First, the SEER database does not provide some crucial information, including detailed smoking, chronic obstructive pulmonary disease, and family history of lung cancer. These factors were considered as the major risk factors of IPLC, whose impacts on the development of SPLC remain to be elucidated. Second, only internal validation was used to test the validity of our risk prediction nomogram, while external validation is regarded as the gold standard[20]. Third, our risk model seems to only have a moderate discrimination. Additional risk factors of developing metachronous SPLC need to be found and added to our risk model, which would promote the accuracy of lung cancer screening and avoid unnecessary radiation. Finally, as a retrospective cohort population, a selection bias in the participants might be present. More large-scale prospective randomized controlled trials are warranted to identify the risk factors of SPLC.

## Conclusion

During the 5-year follow-up, the age-standardized cumulative risk of metachronous SPLC in patients who survived ≥ 2 years after the diagnosis of IPLC increased over time without plateau. The risk prediction nomogram based on histology, age, tumor stage, and radiation treatment can help clinicians to decide whether the survivors of IPLC have a high risk of developing metachronous SPLC and receive low-dose computed tomography surveillance, allowing them to obtain a clinical net benefit in the risk threshold of 0.01 to 0.06. External validation and the addition of other risk factors in the proposed model can potentially improve its validity.

## Supporting information

S1 File Supplementary 0716. (XLSX)

## Abbreviations

APC: annual percentage change
IPLC: initial primary lung cancer
SPLC: second primary lung cancer
CS: condensed stage
